# 早期肺癌术后随访策略需要更多研究证据

**DOI:** 10.3779/j.issn.1009-3419.2018.03.16

**Published:** 2018-03-20

**Authors:** 海泉 陈

**Affiliations:** 复旦大学附属肿瘤医院胸外科 Department of Thoracic Surgery, Fudan University Shanghai Cancer Hospital

非小细胞肺癌的术后复发转移是影响患者长期生存的主要因素。理想的术后随访旨在早期发现复发转移，并能够采取积极的治疗手段。然而，目前探讨非小细胞肺癌术后复发转移及随访模式的文献还很匮乏。国际上对于非小细胞肺癌术后随访策略（包括随访的时间间隔和采用的检查方法）还没有达成共识。比如，美国国立综合癌症网络（National Comprehensive Cancer Network, NCCN）指南推荐对于Ⅰ期-Ⅱ期的非小细胞肺癌患者，术后2年-3年，每6个月行1次胸部CT以及病史采集和体格检查；随后每年1次胸部计算机断层扫描（computed tomography, CT）及病史采集和体格检查^[1]^。而2015年版的中国原发性肺癌诊疗规范推荐的术后患者随访频率为2年内每3个月-6个月随访1次，2年-5年每6个月随访1次，5年后每年随访1次。采用的检查方法包括病史、体检、血生化和血液肿瘤标志物检查、影像学检查和内镜检查等^[2]^。

该论著中，戴亮等回顾性分析了2000年1月-2013年10月北京大学肿瘤医院胸外一科单一医生组连续行解剖性肺叶切除手术的416例Ⅰ期非小细胞肺癌患者的复发转移模式。随访率达到98.7%。存活患者中位随访时间达到71.2个月。随访过程中共有76例患者出现复发转移，包括21例肺转移、20例脑转移、12例骨转移、12例纵隔淋巴结转移、6例肝转移、3例胸膜转移以及2例皮下转移。术后2年内与3年-5年复发转移风险相当。单因素分析发现pT2a以及术前心肺系统合并症是复发预测因子。此外，作者还发现肺、纵隔淋巴结和肝转移多数通过影像学检查发现，而胸膜、脑及骨转移多数是有症状就诊后检查发现。

该研究具有较大的样本量，所选取病例均接受标准肺叶切除及系统性淋巴结清扫，且随访时间长、失访率低，因而研究结果具有一定的意义。该研究的最大特色是分析了特定的复发部位以及复发时间。作者详细描述了哪些部位的复发通常无症状通过检查发现，而哪些部位的复发通常是有症状检查发现。这些研究数据能够增进我们对于早期非小细胞肺癌术后复发模式的理解，进而为随访策略的制定提供理论支持。

关于非小细胞肺癌术后随访策略，目前的Ⅲ期期随机对照临床试验是法国开展的IFCT-0302^[3]^。该研究将完全切除的非小细胞肺癌患者随机分为两组：一组术后随访仅包括临床检查和胸片；另一组术后随访包括临床检查、胸片、胸腹部CT以及气管镜。该研究的初步结果发现两组之间总生存没有差异。然而，鉴于非小细胞肺癌是一个异质性很大的群体，肿瘤大小、分期、病理类型、腺癌亚型、脉管侵犯等因素均会影响复发转移。展望未来，非小细胞肺癌术后个体化随访策略的制定仍需要更多的研究证据。
